# A New Method for *In Vitro* Detection of Bromodeoxyuridine in Serum: A Proof of Concept in a Songbird Species, the Canary

**DOI:** 10.1371/journal.pone.0063692

**Published:** 2013-05-14

**Authors:** Jennifer M. Barker, Thierry D. Charlier, Gregory F. Ball, Jacques Balthazart

**Affiliations:** 1 GIGA Neurosciences, Research Group in Behavioral Neuroendocrinology, University of Liege, Liege, Belgium; 2 Department of Psychological and Brain Sciences, Johns Hopkins University, Baltimore, Maryland, United States of America; Institut National de la Recherche Agronomique-CNRS UMR6175, France

## Abstract

Systemic injection of a thymidine analogue such as bromodeoxyuridine (BrdU) in vertebrates is commonly used to detect and study cell production during development, adulthood, and pathology, particularly in studies of adult neurogenesis. Although researchers are applying this technique to multiple species in various physiological conditions, the rate of BrdU clearance from the serum remains unknown in most cases. Changes in this clearance rate as a function of the species, sex or endocrine condition could however profoundly affect the interpretation of the results. We describe a rapid, sensitive, but simple bioassay for post-injection detection and quantification of BrdU in serum. This procedure was shown to be suitable for determining the length of time a thymidine analogue remains in the bloodstream of one avian species and seems applicable to any vertebrate provided sufficiently large blood samples can be collected. This technique was used to demonstrate that, in canaries, BrdU injected at a dose of 100 mg/kg is no longer available for incorporation into DNA between 30 and 60 min post-injection, a delay shorter than anticipated based on the available literature. Preliminary data suggest a similar fast clearance in Japanese quail and mice.

## Introduction

The injection of thymidine analogues such as bromodeoxyuridine (BrdU) that are incorporated into DNA during its replication continues to be the best and most commonly used technique for investigating proliferating populations of cells *in vivo* [1], [2]. This is particularly true in the neurosciences, where injections of BrdU have been commonly used to assess the rate of neuronal progenitor proliferation both during ontogeny [3] and in adulthood [2],[4]–[6]). In addition, since newly-formed neuronal precursors very often do not undergo many subsequent divisions (in particular during adult neurogenesis), the incorporated BrdU can be used to trace the survival of the new neurons (e.g., [7]–[11]).

Most studies using bromodeoxyuridine (BrdU) to label mitotic cells assume a bioavailability time of approximately 2 h after injection. This is based primarily on early studies that relied on detecting radioactive thymidine or thymidine analogues in tissue of mice [12]–[14], rats [12], and primates [15], [16]. However, there is little information on the persistence in serum of BrdU or other thymidine analogues in other species, despite indirect evidence that this time is not consistent across species. Pregnant rhesus monkeys, for example, clear tritiated thymidine from plasma considerably more rapidly than 2 h [16]. It is therefore possible that there may be species, strain, life stage, or sex differences in clearance rates that could affect the total number of cells labelled and have dramatic effects on the interpretation of results from experiments that include injecting BrdU. Treatments that influence clearance rates could also affect cell counts independently of cell proliferation changes. Multiple studies have for example identified differences in the number of new neurons labelled with BrdU as a function of the sex or endocrine conditions of the subjects (for recent reviews see [17]–[19]). This could reflect true differences in the rate of neurogenesis, but undetected changes in BrdU clearance from the serum could have the same effect.

As research using these analogues advances, determining the clearance rate of BrdU or other thymidine analogues quickly and easily in model organisms of interest is therefore important for the development of appropriate experimental timelines and protocols, for confirming successful injection, and for ensuring that detected differences in proliferation rates are not the result of differential clearance of the thymidine analogue(s) used. The rate at which such molecules are cleared from the circulatory system is unknown for most species, in part because they can be difficult to detect in solution when not bound to DNA.

Injected thymidine analogues can be detected in a variety of different ways, including radioassays [12] or high-performance liquid chromatography (HPLC) [20], [21]. However, the former requires the *in vivo* injection of radioactive (typically tritiated) analogues, appropriate detection equipment, and generating substantial amounts of radioactive waste, while the latter requires specialized equipment specifically calibrated to differentiate the analogue from any endogenous thymidine that may be present. These technical difficulties mean that BrdU concentrations in blood are rarely quantified. We therefore sought to develop a fast, simple and inexpensive method that would allow for detection and quantification of BrdU in serum with the need for minimal amounts of equipment. We present here a bioassay in which we expose living cultured eukaryotic cells to serum from several species injected with BrdU, and then allow the cells to incorporate the BrdU from the serum. The technique then relies on standard immunohistochemical staining techniques. It thus could be used to detect any thymidine analogue that is normally detectable in tissue, injected into any species from which serum can be collected, using any proliferative eukaryotic cell strain that can be cultured. We demonstrate the utility of this technique using HEK293T cells to detect free BrdU in the serum of canaries (*Serinus canaria*). We selected this species because studies of canaries arguably played a key role in initiating the modern era of studies of adult neurogenesis in vertebrates [22], [23]. We also studied a few quail and mice, at various time points after BrdU injection to provide preliminary insight in the generalizability of these findings.

## Materials and Methods

### Animal Subjects

Six adult canaries (*Serinus canaria*, 4 males and 2 females) of mixed-breed origin were obtained from a local breeder (Noorderwijk, Belgium). Two adult female Japanese quail (*Coturnix japonica*) from an in-house breeding colony (University of Liège, Belgium), and three adult C57BL6 mice *(Mus musculus* 2 males and 1 female*)* from the central breeding colony at the University of Liège, Belgium were also used. All experimental procedures complied with the relevant Belgian laws concerning the Protection and Welfare of Animals and the Protection of Experimental Animals. Experimental protocols were approved by the Ethics Committee for the Use of Animals at the University of Liège (protocol number 926).

### BrdU Administration and Sample Collection

A single intraperitoneal injection of BrdU (100 mg/kg, dissolved in sterile 0.9% saline) was given to each animal. We then collected in microcentrifuge tubes peripheral blood samples (via brachial vein puncture in birds, tail vein puncture in mice) 15 min, 30 min, 60 min and 120 min after injection. Four hours (240 min) after injection, trunk blood samples were taken after animals were deeply anesthetized and decapitated. Blood was allowed to clot overnight, centrifuged at 10,000 *g* for 10 minutes, and the serum was collected and stored at −20°C until analysis.

### Cell Culture

HEK293T cells were cultured on glass coverslips (12 mm diameter stored in 24 well culture plates) in Dulbecco’s modified Eagle’s medium (Invitrogen) containing 10% fetal bovine serum (DMEM+FBS, 500 µl) at 37°C for 24 h. Cultures were then rinsed and incubated at 37°C in DMEM+FBS for another 4 h with the addition of either 10 µL of a known concentration (from 0 mg/mL to 3 mg/mL) of BrdU (dissolved in pooled serum from uninjected birds, in normal horse serum, or in 0.9% saline), or 10 µL of serum from a subject previously injected with BrdU. The cultured cells were then immediately processed with immunohistochemistry against BrdU.

### Immunohistochemical Staining

Plated cells were rinsed with 0.01 M phosphate-buffered saline (pH 7.4; PBS), fixed by incubation in 4% formaldehyde in PBS for 20 min, and rinsed three times in PBS. Cells were then incubated with 0.6% hydrogen peroxide in PBS, rinsed, incubated 10 min in 2N HCl followed by 5 min in 0.1 M sodium borate buffer (pH 8.5), rinsed again, and blocked 8 min in 10% normal horse serum in PBS with 0.02% Triton-X100 (PBS-T) followed by incubation for 2 h at 4°C with monoclonal rat anti-BrdU primary antibody (AbD Serotec, 1∶1000) in PBS-T. Plates were rinsed three times in PBS, followed by incubation for 30 min at room temperature in donkey anti-rat biotinylated secondary antibodies (Jackson Immunoresearch, 1∶2000) in PBS-T. After another three rinses in PBS, plates were incubated 30 min at room temperature with an avidin-biotin complex (Vectastain Elite kit, Vector Laboratories). Plates were rinsed in PBS and the label visualized by incubation at room temperature for 8 min with 3,3′-diaminobenzidine (DAB; 0.04% DAB with 0.012% H_2_O_2_ in PBS). The DAB solution was rinsed off with PBS, and plates were mounted onto glass microscope slides with an aqueous mounting medium (glycerine with phenol).

### Image Analysis

**Figure 1:**
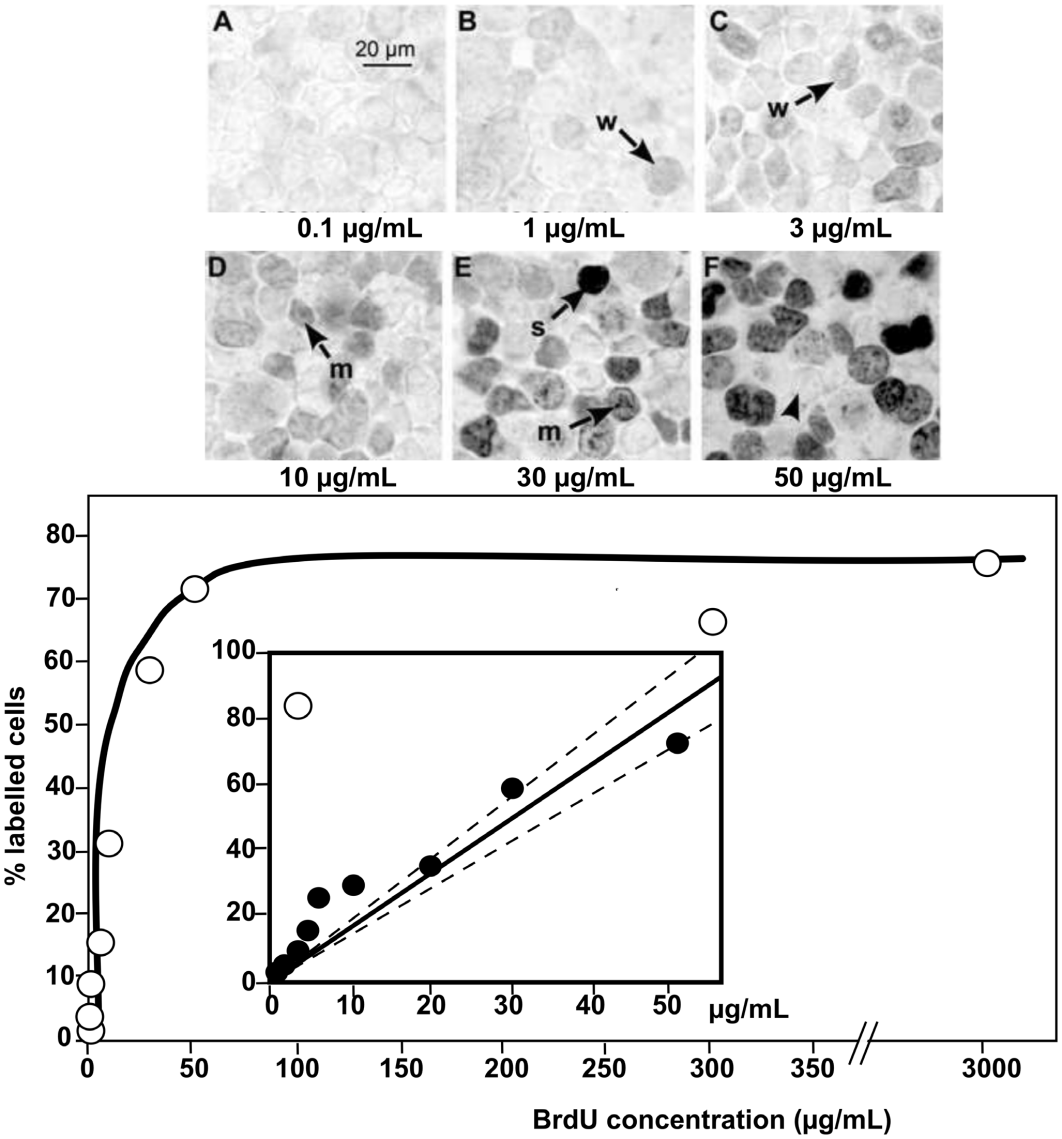
Dose-response curve of labelling index after incubation of HEK293T cells with BrdU. (A–F) Cultured cells stained for BrdU, after incubation with BrdU at different concentrations (as indicated at the bottom of each image). Images were consistently modified to enhance contrast and remove colour, and are all shown to the same scale. (A) At very low concentrations, very few cells were labelled even weakly. (B–F) As BrdU concentration increased, the proportion of visible cells that were weakly-labelled (w), moderately-labelled (m), or strongly-labelled (s) increased. Every culture plate also contained some unlabelled cells (arrowhead). (G) Total numbers of labelled cells (weakly+moderately+strongly) reached a plateau with BrdU concentrations above 50 µg/mL; note the break in the x-axis scale**.** At lower concentrations, however, (inset) there was a strong linear relationship between BrdU concentration and the total number of labelled cells (Y = 1.651 X, line forced through [0,0]; confidence interval for slope = 1.403 to 1.898, r = 0.9606, p<0.0001). Dashed lines show 95% confidence interval around the (solid) line of best fit.

Cells were counted in digital photomicrographs (two per sample) taken under a 40× objective (Olympus microscope, Scion Corporation camera, ImageJ software) after processing in a standardized manner for analysis using Preview software (Apple Inc.) to uniformly maximize contrast and desaturate the images. We counted three classes of labelled cells in the resulting images: strongly labelled (nucleus entirely black), moderately labelled (nucleus grey with some black spots), and weakly labelled (nucleus grey with no black spots) (Fig. 1 A–F). We also counted the total number of cells in images with eliminated highlights and uniformly enhanced contrast to visualize cell membranes; we only counted cells (labelled or not) with nuclei entirely visible within the field of view. The percentage of total visible cells that were labelled was calculated for each image; data are reported as the average of the two images for each sample.

## Results

### Calibration Curves and Threshold Determination

We first exposed cultured cells to decreasing known concentrations of BrdU, to determine the sensitivity of the assay. The presence of BrdU was detectable for added concentrations from 3 mg/mL down to 5 µg/mL, and weakly detectable at 3 µg/mL (see Fig. 1A–F). No difference in the density of labelled cells was observed as a function of whether BrdU was diluted in unlabelled bird serum, normal horse serum, or 0.9% saline. We therefore used 0.9% saline as the standard diluent for the dilution curve run alongside all serum samples.

### Relationship between Labelling Index and BrdU Concentration

We found moderately- and strongly-labelled cells only in cultures given BrdU at concentrations above 10 µg/mL (Fig. 1D–F). However, the number of these cells (20–60% of the total) did not clearly relate to the BrdU concentrations in this range: saturation was rapidly observed for increasing concentrations (data not shown). When weakly-labelled cells were included in the total counts, labelled cells were reliably observed at BrdU concentrations down to 3 µg/mL, and the percentage of labelled cells was linearly related to the BrdU concentration for concentrations up to 50 µg/mL (% labelled cells = (1.651±0.115)× BrdU conc in µg/mL, p<0.0001, regression forced through (0,0); see inset of Fig. 1G). The total counts of labelled cells then reached a plateau at higher concentrations (Fig. 1G). All data are thus presented as the total of all types of labelled cells (weakly, moderately, and strongly labelled).

### BrdU Clearance from Serum *in vivo*

**Figure 2:**
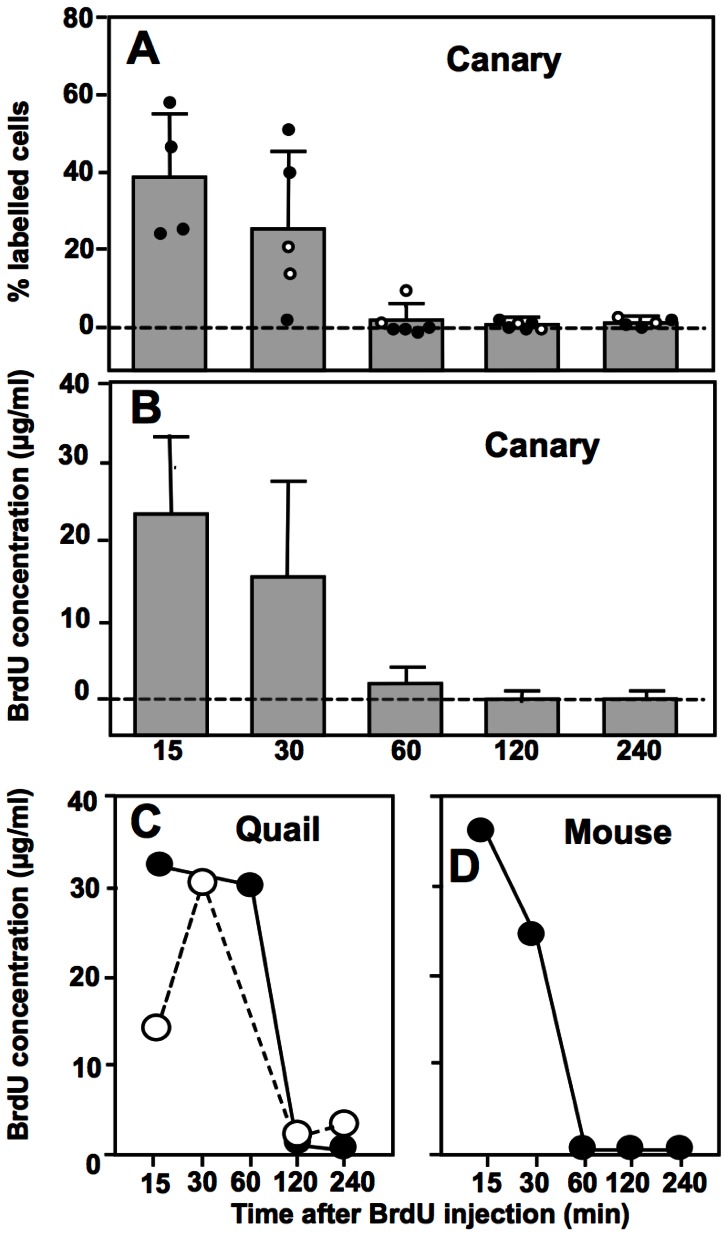
Labelling indices and deduced BrdU concentrations after incubation of HEK293T cells with serum from BrdU-injected animals. (A). Percentage (means ± SD) of the total numbers of cells visible in culture that were immunoreactive for BrdU after incubation with serum collected from adult canaries at various times after BrdU injection. All individual male (black spots) and female (open circles) data points are represented in the figure. (B) Average concentrations (means ± SD) of BrdU in these canary samples based on the calibration curve illustrated in the inset of Figure 1G. (C–D) Concentrations of BrdU in blood samples collected in two female Japanese quail (C) or one male mouse (D) at various times after a single BrdU injection.

No labelled cells were detected in cultures that had been treated with serum collected at 120 or 240 min after BrdU injection in any of the three species considered. At these two late time points, BrdU concentration in serum was thus below 3 µg/mL and presumably not sufficient to label cells *in vivo* in any of the species studied (Fig. 2).

In canaries, at the 15 and 30 min time points, the average percentage of labelled cells (weakly+moderately+strongly) ranged between 20 and 40% of the total, although the individual variation in this percentage was relatively large especially at the 30 min time point (Fig. 2A). Based on the calibration curve presented in the inset of Figure 1, these percentages of labelled cells were shown to correspond to BrdU concentrations ranging between 10 and 25 µg/mL (Fig. 2B) and definitely below 50 µg/mL. The serum concentrations of BrdU obtained soon after the injection were thus well below the theoretical maximum blood concentration of 1 mg/mL. Indeed, assuming that approximately 10% of overall body weight is blood and that all BrdU enters the blood, an injection of 100 mg BrdU per kg body weight should correspond to a total injection of approximately 100 mg per 100 mL blood volume, i.e. a concentration of 1 mg/mL. Actual concentrations detected at 15 min post-injection were thus only 5% of this maximal theoretical value indicating either a limited absorption or rapid catabolism.

Labelling of cells was then nearly absent from the canary samples collected 60 minutes after injection indicating a lack of BrdU in most of these sera (concentration below 3 µg/mL in all samples except one 60 min sample). No evidence of a sex difference in BrdU concentrations could be detected here in canaries (compare open and filled circles in Fig. 2A) but additional work should obviously be performed to confirm this impression.

A few serial samples were also able to be collected and assayed for BrdU in quail and mouse. These results indicate that the present technique should be applicable to a broad range of species and suggest a similar rapid clearance of BrdU from the blood of these species (Fig. 2C–D).

## Discussion

We report here a fast, simple, and inexpensive method for detecting thymidine analogues in serum after injection in various species by making use of cultured, dividing cells as detectors. Using this technique we show here that in canaries (and preliminary data indicate that it is also highly likely to be the case in Japanese quail and mice), unbound BrdU is cleared from circulating blood within 2 h after intra-peritoneal injection. This is reasonably consistent with the bioavailability data previously presented for other species [12]–[16], [20], [21]. However, after incubation with canary (Fig. 2A–B; and possibly mouse, Fig. 2D) serum samples collected 60 minutes after BrdU injection, labelled cell counts were already at a similar low level. This suggests that in this (these) species the clearance rate of BrdU from serum may be faster than typically claimed, although this does not preclude the possibility that BrdU may be sequestered within, and available at low levels for incorporation in, other tissues including the brain. One quail sample still contained a high BrdU concentration (around 30 µg/mL) at 60 min post-injection (Fig. 2C) suggesting lower clearance in this species but more work would obviously be needed to confirm this single observation. Species differences in BrdU bio-availability could relate to body size (250–300 g in quail as compared to 15–30 g in mice or canaries), to metabolism, or to species differences in the efficiency either of active pyrimidine transport or of diffusion from the intraperitoneal cavity into the circulatory system. Additional species would need to be tested to further explore these possibilities.

Regardless of the underlying mechanism, that species differences may exist in the rate at which BrdU enters and/or is removed from circulation is important to note, as such differences could bias comparisons across species when counts of labelled cells in tissue are compared. All other factors being equal, including *in vivo* cell proliferation rates, a species with faster clearance of BrdU (or other thymidine analogue) would appear to have fewer labelled cells in tissue after injection than a species with a slower clearance rate, simply due to BrdU not being available for uptake by cells for the same length of time.

The bioassay presented here can be used to measure BrdU or any other thymidine analogue detectable by immunohistochemistry (e.g. iodo-deoxyuridine [IdU]: [1], [24]; chloro-deoxyuridine [CldU]:[25]–[28]; ethynyl-deoxyuridine [EdU]: [29], [30]) in serum at multiple times after injection. It would allow for the generation of a time course of BrdU availability in the circulation for any species, and after any sort of treatment, with the advantages of being rapid and not requiring much specialized equipment. Injection and serum collection can be completed in a single day, while cell culture incubation and immunohistochemical staining can be done on the following day. Although assays were performed here on sera obtained from blood that was allowed to clot overnight, there is no reason why the same technique could not be applied on sera centrifuged within minutes of collection. Furthermore, we used here HEK293T cells as *in vitro* detectors for BrdU but these could be readily substituted with any cell line that multiplies efficiently in culture. It should also be noted that cells were grown on sterile glass coverslips placed in 24 well plates. If a cell culture facility is not available in a laboratory interested by this technique, a collaboration with a laboratory that has this technique available could thus be easily developed. Once cells have been grown in sterile conditions on the coverslips, the wells containing these cultures can easily be covered and transported to a classical histology laboratory where the 4 hour incubation with samples containing BrdU and immunohistochemical staining can be performed in conditions that are no longer sterile. This is actually what was done here (original culture in sterile conditions and remaining procedures in a standard histology facility). If detailed analysis is not required and only a semi-quantitative assessment is needed, a single blood sample can be taken at 15–30 min after injection, and a simple qualitative measure (presence/absence) can be obtained as soon as the stained culture plates are mounted on slides. The entire procedure from injection to final data analysis can therefore be realistically completed in less than 2 days.

In conclusion, the method presented here provides a proof of concept for a technique to investigate the rate of BrdU clearance in canaries, a species in which pioneering studies of adult neurogenesis were initiated 30 years ago [22], [23]. This method can be used to estimate the length of time after injection for which BrdU is available for incorporation into new cells in any given vertebrate species. It would allow for the investigation of differences in pyrimidine uptake and clearance rates between sexes, or among different developmental stages. It can also constitute a rapid first step into investigations of experimental treatment effects in BrdU uptake by, or clearance from, the serum, particularly treatments that may affect pyrimidine metabolism. Finally, this method could be easily adapted for detection of any thymidine analogue for which an appropriate immunohistochemical detection method is available for use in fixed cells. We have demonstrated the utility of this technique using HEK293T cells to detect free BrdU in the serum of canaries, quail, and mice at various time points after BrdU injection. There is, however, no apparent barrier to using it for detection of any thymidine analogue normally detectable in tissue, injected into any species from which serum can be collected, using any proliferative eukaryotic cell strain that can be cultured.
